# Cu-Doping Induced Structural Transformation and Magnetocaloric Enhancement in CoCr_2_O_4_ Nanoparticles

**DOI:** 10.3390/nano15141093

**Published:** 2025-07-14

**Authors:** Ming-Kang Ho, Yun-Tai Yu, Hsin-Hao Chiu, K. Manjunatha, Shih-Lung Yu, Bing-Li Lyu, Tsu-En Hsu, Heng-Chih Kuo, Shuan-Wei Yu, Wen-Chi Tu, Chiung-Yu Chang, Chia-Liang Cheng, H. Nagabhushana, Tsung-Te Lin, Yi-Ru Hsu, Meng-Chu Chen, Yue-Lin Huang, Sheng Yun Wu

**Affiliations:** 1 Department of Physics, National Dong Hwa University, Hualien 974301, Taiwan; 2 National Synchrotron Radiation Research Center, Hsinchu 30076, Taiwan; 3 Prof. C.N.R. Rao Centre for Advanced Materials, Tumkur University, Tumkur 572103, India; 4 Department of Mechanical and Systems Engineering, National Atomic Research Institute, Taoyuan 325207, Taiwan; 5 Department of Applied Science, National Taitung University, Taitung 950, Taiwan

**Keywords:** CoCr_2_O_4_ nanoparticles, Cu doping, magnetocaloric effect (MCE), Curie temperature, magnetic entropy change, magnetic refrigeration, second-order phase transition

## Abstract

This study systematically investigates the impact of Cu^2+^ doping on the structural, magnetic, and magnetocaloric properties of Cu*_x_*Co_1−*x*_Cr_2_O_4_ nanoparticles synthesized via a solution combustion method. Cu incorporation up to *x* = 20% induces a progressive structural transformation from a cubic spinel to a trigonal corundum phase, as confirmed by X-ray diffraction and Raman spectroscopy. The doping process also leads to increased particle size, improved crystallinity, and reduced agglomeration. Magnetic measurements reveal a transition from hard to soft ferrimagnetic behavior with increasing Cu content, accompanied by a notable rise in the Curie temperature from 97.7 K (*x* = 0) to 140.2 K (*x* = 20%). The magnetocaloric effect (MCE) is significantly enhanced at higher doping levels, with the 20% Cu-doped sample exhibiting a maximum magnetic entropy change (−Δ*S_M_*) of 2.015 J/kg-K and a relative cooling power (RCP) of 58.87 J/kg under a 60 kOe field. Arrott plot analysis confirms that the magnetic phase transitions remain second-order in nature across all compositions. These results demonstrate that Cu doping is an effective strategy for tuning the magnetostructural response of CoCr_2_O_4_ nanoparticles, making them promising candidates for low-temperature magnetic refrigeration applications.

## 1. Introduction

Transition metal oxides with spinel structures, particularly cobalt chromite (CoCr_2_O_4_), have attracted significant attention due to their diverse applications, including catalysis, magnetic materials, and energy storage systems [1,2,3,4,5]. The CoCr_2_O_4_ spinel structure, known for its high thermal stability and robust mechanical properties, provides a versatile platform for doping with various metal ions, enabling the fine-tuning of magnetic, structural, and electronic properties for targeted applications [6,7,8,9,10].

Among the transition metals, copper (Cu) has emerged as a promising dopant due to its unique electronic configuration (3d^10^4s^1^) and relatively similar ionic radius compared to Co^2+^. Copper doping has been demonstrated to induce substantial changes in spinel oxides’ magnetic interactions, structural order, and phase transitions. In CoCr_2_O_4_, these alterations are of particular interest due to the material’s intrinsic ferrimagnetic behavior, which can be enhanced or modified through doping to optimize its properties for applications such as magnetic refrigeration, spintronics, and magnetic memory devices [1,2,3,4,5]. Cu doping affects both the magnetic and morphological properties of CoCr_2_O_4_ nanoparticles. Structurally, substituting Co^2+^ with Cu^2+^ can lead to lattice distortions due to the difference in ionic radii, which influences particle size, shape, and distribution. These structural modifications can reduce particle agglomeration and enhance dispersion, as observed in other doped systems. In terms of magnetic properties, Cu doping typically weakens the overall magnetic interactions by substituting non-magnetic Cu^2+^ for magnetic Co^2+^ ions, reducing the saturation magnetization (*M_S_*) and Curie temperature (*T_C_*) [11]. However, the presence of Cu ions can also increase the magnetocaloric effect (MCE), a phenomenon where magnetic entropy changes in response to applied magnetic fields, making doped CoCr_2_O_4_ a potential candidate for magnetic refrigeration technology.

Beyond the well-known positive magnetocaloric effect (MCE) observed in materials like Gd-based alloys, recent research has uncovered the occurrence of negative MCE in a variety of advanced materials systems. Negative MCE arises when the application of a magnetic field leads to an increase in magnetic entropy, typically due to field-induced spin disorder or the suppression of antiferromagnetic correlations, rather than spin alignment. This intriguing behavior has been observed in intermetallic compounds, perovskite oxides, and, more recently, certain superconducting hybrids and nanocomposites, where strong spin–lattice coupling and quantum fluctuations play a significant role [12]. These findings significantly extend the functional range of MCE materials beyond cryogenic regimes and enable cooling applications in unconventional temperature windows. Thus, a comprehensive understanding of both structural phase transitions and magnetic ordering phenomena is critical in engineering MCE properties and advancing next-generation solid-state refrigeration technologies across diverse materials platforms.

Modifying magnetic phase transitions in spinel chromites through ionic substitution has emerged as a promising strategy for tuning their magnetic and magnetocaloric properties. Previous studies have explored various dopants to understand how subtle changes in cation composition influence magnetic ordering. For instance, Ptak et al. [13] examined the effect of nickel incorporation in CoCr_2_O_4_ and reported that the partial substitution of Co^2+^ with Ni^2+^ resulted in marginal reductions in both the Curie temperature (*T_C_*) and the spiral spin transition temperature (*T_S_*), indicating a suppression of long-range magnetic order. Similarly, Ghosh et al. [14] demonstrated that doping with Gd^3+^ ions led to a significant enhancement in saturation magnetization (*M_S_*) at 5 K. This improvement was attributed to both the increased magnetic moment introduced by Gd^3+^ and a decrease in coercivity. Additionally, the reduction in particle size at higher Gd concentrations introduced surface-related phenomena such as spin canting, pinning, and disorder, which further affected the magnetic anisotropy. The influence of alkaline earth metal doping has also been explored. Kamran et al. [15] reported a progressive decrease in both *T_S_* and *T_C_* with increasing Mg^2+^ content in CoCr_2_O_4_ nanoparticles, underscoring the impact of ionic substitution on magnetic phase stability. Similarly, Dutta et al. [9] documented negative magnetization in CoCr_2_O_4_ nanoparticles, reinforcing the role of surface spin disorder. Li et al. [16] explored the influence of crystallite size on the magnetocaloric properties of CoCr_2_O_4_, revealing that the nanocrystalline form exhibited significantly enhanced magnetic behavior and entropy change compared to its bulk counterpart, thereby underscoring the critical role of size effects in optimizing MCE performance for practical applications. Further insights into doped systems were provided by Gulkesen et al. [17], who investigated Fe-doped CoCr_2_O_4_ and reported a peak magnetic entropy change (−Δ*S_M_*) of 0.88 J/kg-K for the undoped sample. Additionally, Ram Kumar et al. [18] examined the magnetocaloric performance of Co(Cr_0_._95_Fe_0_._05_)_2_O_4_ and identified a maximum cooling power of 13 J/kg, further demonstrating the tunability of MCE in doped spinel systems.

Despite these advancements, the influence of Cu^2+^ doping on the structural evolution and magnetocaloric performance of CoCr_2_O_4_ remains underexplored. While sporadic studies have addressed Cu-substituted spinels, a systematic investigation across a broad concentration range, particularly using the solution combustion synthesis technique, has yet to be reported. This method, known for its ability to produce highly homogeneous nanoparticles with fine crystallinity, offers significant advantages over conventional solid-state and sol–gel techniques, which often suffer from non-uniform doping and particle agglomeration [19,20].

In the present study, CoCr_2_O_4_ nanoparticles doped with varying concentrations of Cu (*x* = 0, 5, 10, 15, and 20%) were synthesized via the solution combustion method. The structural, morphological, and compositional characteristics were thoroughly analyzed using X-ray diffraction (XRD), Raman, scanning electron microscopy (SEM), transmission electron microscopy (TEM), and energy-dispersive X-ray spectroscopy (EDX). The magnetic behavior and magnetocaloric properties were systematically evaluated through temperature-dependent magnetization measurements. This work provides a new understanding of how Cu incorporation modulates the magnetostructural coupling and entropy changes in CoCr_2_O_4_, offering valuable insights for the development of advanced materials for magnetic refrigeration and spintronic applications.

## 2. Experimental Details

### 2.1. Synthesis Procedure

Copper-doped cobalt chromite (Cu-Co-Cr) nanoparticles were synthesized via the solution combustion synthesis (SCS) method, as systematically illustrated in Scheme S1a–f (Supporting Information). This process involves a redox-driven, self-sustaining reaction between metal nitrates and organic fuels, resulting in rapid nanoparticle formation under relatively mild conditions. In the first step (Scheme S1a, Supporting Information), a precursor solution was prepared by dissolving stoichiometric amounts of cobalt nitrate tetrahydrate [Co(NO_3_)_2_·4H_2_O], copper nitrate trihydrate [Cu(NO_3_)_2_·3H_2_O], and chromium nitrate nonahydrate [Cr(NO_3_)_3_·9H_2_O] in deionized water. These metal salts acted as oxidizing agents and sources of Co, Cu, and Cr cations. To drive the combustion reaction, organic fuels—urea [CO(NH_2_)_2_] and glucose [C_6_H_12_O_6_]—were added in a 1:1 molar ratio relative to the total moles of metal nitrates. Urea supports gas evolution and the flame temperature, while glucose improves thermal reactivity and acts as a carbon-based fuel. The solution was then subjected to vigorous stirring using a magnetic stirrer for approximately 30–45 min at room temperature to ensure complete dissolution and achieve a uniform, transparent, and homogeneously mixed precursor solution (Scheme S1b, Supporting Information). This homogeneous mixing is essential in ensuring an even distribution of metal and fuel ions, leading to uniform combustion behavior. Next, the homogeneous precursor solution was transferred to a heat-resistant glass container and carefully placed in a preheated muffle furnace maintained at 450 °C (Scheme S1c, Supporting Information). Within 20–25 min, a rapid and self-sustained exothermic combustion reaction initiated spontaneously due to the redox interaction between the oxidants and fuels. The reaction was accompanied by the evolution of large volumes of gaseous byproducts (e.g., N_2_, CO_2_, H_2_O), leading to the formation of a highly porous, foamy, ash-like solid residue with an expanded volume and low bulk density. Following the initial combustion, the as-synthesized material was subjected to a calcination step at 600 °C for 2 h in air, using a programmable muffle furnace (Scheme S1d, Supporting Information). This step plays a crucial role in removing residual carbonaceous and organic species, decomposing intermediate phases, and promoting crystallization into the spinel Cu-doped CoCr_2_O_4_ structure. Calcination also improves thermal stability and phase purity. After calcination, the product was allowed to cool to room temperature and then manually ground using an agate mortar and pestle to break down agglomerates and obtain a uniform, fine nanopowder (Scheme S1e, Supporting Information). This grinding process enhances the surface area and facilitates subsequent characterization and application. The final product, shown in Scheme S1f (Supporting Information), consisted of a fine, homogeneous nanopowder of copper-doped cobalt chromite, suitable for structural, morphological, and functional property analyses.

### 2.2. Characterization Techniques

The morphology and elemental composition of the synthesized nanoparticles were characterized using field-emission transmission electron microscopy (FE-TEM, JEM-F200, JEOL Ltd., Tokyo, Japan), field-emission scanning electron microscopy (FE-SEM; JEOL JSM-7000F, JEOL Ltd., Tokyo, Japan) coupled with energy-dispersive X-ray spectroscopy (EDX). X-ray diffraction (XRD) analysis was performed using a Rigaku MiniFlex 600 diffractometer equipped with Cu Kα radiation (λ = 1.5406 Å) to identify the crystalline phases and calculate structural parameters. Raman spectroscopy was conducted with a Leica micro-Raman spectrometer employing a 532 nm excitation laser to investigate vibrational modes and confirm structural features across different temperature ranges. The magnetic properties and magnetocaloric performance were evaluated using a superconducting quantum interference device vibrating sample magnetometer (SQUID-VSM; Quantum Design, EverCool system) to capture magnetization behavior as a function of the temperature and applied magnetic field.

## 3. Results and Discussion

### 3.1. Morphological Characterization of Cu_x_Co_1−x_Cr_2_O_4_ Nanoparticles

**Figure 1:**
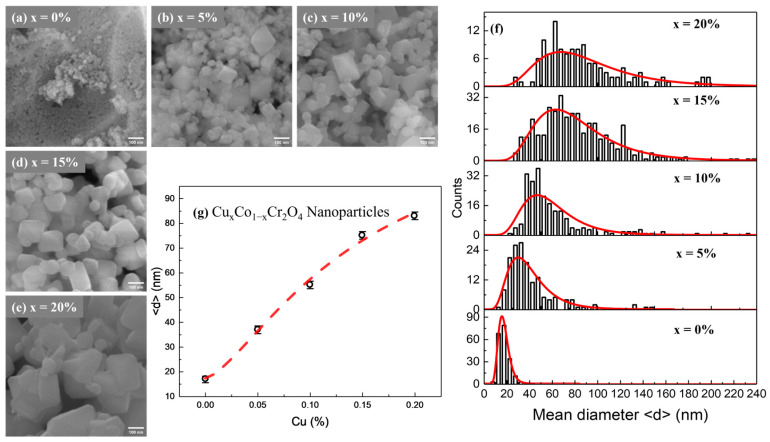
(**a**–**e**) FE-SEM images of Cu*_x_*Co_1−*x*_Cr_2_O_4_ nanoparticles. (**f**) Histogram diagram based on FE-SEM images of Cu*_x_*Co_1−*x*_Cr_2_O_4_ nanoparticles. (**g**) Variation in mean diameter as a function of Cu concentration for Cu*_x_*Co_1−*x*_Cr_2_O_4_ nanoparticles.

Scanning electron microscopy (SEM) was employed to investigate the surface morphology and particle size distribution of CoCr_2_O_4_ nanoparticles doped with varying Cu concentrations (*x* = 0, 5, 10, 15, and 20%). The representative SEM images are shown in Figure 1a–e. Undoped CoCr_2_O_4_ (Figure 1a) shows highly agglomerated, irregularly shaped nanoparticles with relatively small primary particles [21,22]. Upon doping with Cu, notable changes in morphology are observed. At 5% Cu doping, the nanoparticles begin to exhibit more defined cubic-like shapes with moderate agglomeration, indicating the influence of Cu^2+^ ions on crystal growth [22]. With further increases in Cu concentration to 10% and 15%, the particles become more faceted and grow in size, forming compact yet well-defined structures. The 20% Cu-doped sample also shows a uniform, highly crystalline morphology with minimal porosity and relatively larger cubic particles compared to the undoped sample. Figure 1f presents particle size distribution histograms fitted with log-normal curves for each composition. The distributions widen and shift toward higher diameters as the Cu content increases, confirming the size enhancement observed in Figure 1g. Contrary to size reduction trends often observed with certain dopants, here, the mean particle diameter increases progressively with Cu doping, from approximately 17 nm for undoped CoCr_2_O_4_ to over 83 nm for the 20% Cu-doped sample. This increase may be attributed to the enhanced grain growth promoted by Cu incorporation during combustion synthesis.

Additionally, Cu doping appears to reduce particle agglomeration and improve dispersion across the micrographs. The clearer boundaries and defined crystal facets at higher doping levels suggest that Cu plays a key role in modifying grain boundary energy and promoting uniform crystal growth. In summary, SEM analysis confirms that Cu doping significantly influences the morphological features of CoCr_2_O_4_ nanoparticles, leading to increased particle size, improved shape definition, and reduced agglomeration. These enhancements in microstructural properties are expected to play a pivotal role in determining the material’s magnetic and functional performance in advanced applications such as magnetic refrigeration.

Energy-dispersive X-ray spectroscopy (EDX) analysis was performed to verify the elemental composition of the Cu_x_Co_1−x_Cr_2_O_4_ nanoparticles. For the undoped sample (*x* = 0), only Co and Cr elements were detected, confirming the pure CoCr_2_O_4_ spinel structure (Figure S1a, Supporting Information). With the introduction of Cu doping (Figure S1b–e, Supporting Information), the EDX spectra revealed the presence of Cu in addition to Co and Cr, validating successful Cu incorporation into the nanoparticles. The EDX results confirmed the absence of other impurities, except for trace amounts of gold (Au) and silicon (Si), attributed to the gold sputter coating and the silicon substrate used during the analysis [23]. These peaks were consistent across all samples and did not interfere with the interpretation of the elemental composition of the synthesized nanoparticles.

### 3.2. Crystal Structural Characterizations of Cu_x_Co_1−x_Cr_2_O_4_ Nanoparticles

**Figure 2:**
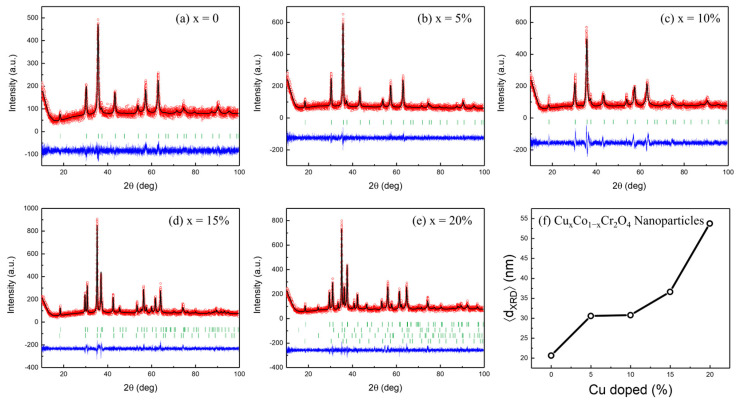
(**a**–**e**) The XRD patterns of Cu*_x_*Co_1−*x*_Cr_2_O_4_ (*x* = 0, 5, 10, 15, and 20%) nanoparticles. (**f**) Average crystallite size $\langle d_{XRD}\rangle$ (nm) obtained from the Williamson–Hall plot as a function of Cu content *x*.

The crystal structure of Cu_x_Co_1−x_Cr_2_O_4_ nanoparticles was analyzed via the Rietveld refinement of X-ray diffraction (XRD) data using the FullProf Suite software (v5.10, January 2023). Figure 2a–e present the refined patterns for doping concentrations of *x* = 0 to 20%, revealing distinct structural trends and phase evolution with increasing Cu content. For the undoped (*x* = 0) and lightly doped samples (*x* ≤ 10%), the diffraction patterns are well indexed to the cubic spinel structure with space group *Fd*-3*m*, consistent with JCPDS number 0801668 [24]. The refinement residuals were minimal in this range, confirming a single-phase spinel structure with high crystallinity. Notably, the gradual shift in diffraction peaks toward lower angles with increasing Cu concentration suggests lattice expansion, attributed to the replacement of Co^2+^ ions (0.74 Å) with slightly larger Cu^2+^ ions (0.73 Å), subtly altering the lattice parameters, as summarized in Table 1.

To probe the microstructural evolution, Williamson–Hall (W–H) analysis was employed to extract both the average crystallite size $\langle d_{XRD}\rangle$ and micro-strain (ε) using the relation [25]:

(1) $$\beta_{T}=\frac{1}{\cos\theta }\left(\frac{k\lambda }{\langle d_{XRD}\rangle }+4\varepsilon \sin\theta \right)$$

where *β_T_* represents the full width at half maximum (FWHM) of the XRD peaks, *θ* is the diffraction angle, k is the Scherrer constant (0.9), λ is the wavelength of the X-rays used (1.54 Å), and ε is the micro-strain. The Williamson–Hall plot was obtained from the XRD diffractogram of Cu*_x_*Co_1−*x*_Cr_2_O_4_ nanoparticles (*x* = 0, 5, 10, 15, and 20%), as shown in Figure S2a–e (Supporting Information). The size vs. strain plots derived from this model showed a systematic increase in crystallite size (Table 1) and a variation in strain at intermediate doping levels, suggesting localized lattice distortions due to dopant incorporation.

As Cu doping increased to *x* = 15% (Figure 2d), the XRD profile exhibited additional peaks that could no longer be indexed to a single cubic phase. Dual-phase refinement was thus carried out, revealing the coexistence of two spinel phases: the dominant *Fd*-3*m* cubic structure and a minor tetragonal spinel phase indexed to *I*4_1_/*amd* [26,27]. The multiphase nature at this concentration implies a structural distortion threshold induced by excess Cu^2+^ incorporation. At *x* = 20% (Figure 2e), a pronounced phase transition occurs. The diffraction pattern is dominated by peaks corresponding to a trigonal corundum structure with space group *R*-3*c*, marking a complete transformation from cubic to trigonal symmetry. This structural transition significantly alters the crystal field environment and suggests a breakdown of the conventional spinel framework at high Cu loading. The refinement confirmed the predominance of the *R*-3*c* phase, with a significant reduction in the original *Fd*-3*m* content.

Figure 2f and Table 1 collectively illustrate the structural evolution of Cu*_x_*Co_1−*x*_Cr_2_O_4_ nanoparticles with increasing Cu doping. Up to *x* = 0.05, the lattice parameter follows Vegard’s law, indicating uniform substitution. However, beyond this point, a distinct non-monotonic trend emerges: the lattice contracts to 8.308 Å at *x* = 0.10, sharply expands to 8.455 Å at *x* = 0.15, and then relaxes slightly to 8.347 Å at *x* = 0.20. This behavior signals the onset of a structural instability driven by the competing elastic responses of the parent cubic (*Fd*-3*m*) spinel and emergent tetragonal (*I*4_1_/*amd*) domains, nucleated by Jahn–Teller (JT) active Cu^2+^ ions.

Quantitative modeling using the Uniform Deformation Energy Density Model (UDEDM) and Williamson–Hall plots reveals that micro-strain (ε) peaks at *x* = 10% (0.384%), corresponding to an elastic energy density of ~1.48 MJ/m^3^—indicating the critical strain limit before the lattice accommodates distortion via symmetry lowering. This elastic threshold marks the transition from a single-phase cubic matrix to a regime of phase coexistence, where the local elongation of CuO_6_ octahedra relieves stress and gives rise to tetragonal nanodomains embedded within the cubic host.

This symmetry breaking is a hallmark of a cooperative Jahn–Teller effect. As Cu^2+^ (3d^9^) replaces Co^2+^ (3d^7^, high-spin) in the octahedral B-sites, orbital degeneracy in the e_g_ level is lifted, leading to the elongation of axial Cu–O bonds and a distortion of the local octahedral field. The interconnected nature of corner-sharing octahedra propagates these distortions collectively, resulting in a long-range symmetry reduction from cubic to tetragonal. This transition is supported by the observed asymmetric broadening and eventual splitting of the (311) and (440) XRD reflections, as well as a systematic red shift and mode emergence in the Raman spectra, notably in the E_g_ and A_1g_ bands, indicative of lower lattice symmetry and expanded metal–oxygen coordination (see Section 3.4).

These results are in line with the crystallographic behavior documented for pure CuCr_2_O_4_ [27], who reported the stabilization of a well-defined tetragonal *I*4_1_*/amd* structure, characterized by *c*/*a* ≈ 1.14 and distinct axial vs. equatorial Cu–O bond lengths (2.35 Å vs. 2.00 Å, respectively). The local distortion correlates with a softened Eg breathing mode near 490 cm^−1^, arising from static JT activity. Although our Cu concentration does not fully reach the endmember characteristics, the inflection near *x* ≈ 0.15 in both lattice strain and diffraction asymmetry aligns closely with the previously reported [27] percolation threshold for cooperative JT ordering. The structural distortion leads to enhanced magnetic anisotropy and weak ferrimagnetism below 125 K, which provides a compelling framework for interpreting our own magnetic data. In particular, the coercivity peak and anomalies in the magnetocaloric response with higher Cu content may reflect the gradual resolution of exchange frustration via orbital reordering and local symmetry breaking. Collectively, these findings confirm that Cu doping induces a progressive tetragonal distortion via a cooperative Jahn–Teller mechanism, which becomes long-range beyond *x* ≈ 0.15, consistent with both our crystallographic and spectroscopic observations and the broader mechanistic trends established for Cu-based chromites.

### 3.3. TEM and HRTEM Characterization of Cu-Doped CoCr_2_O_4_ Nanoparticles

**Figure 3:**
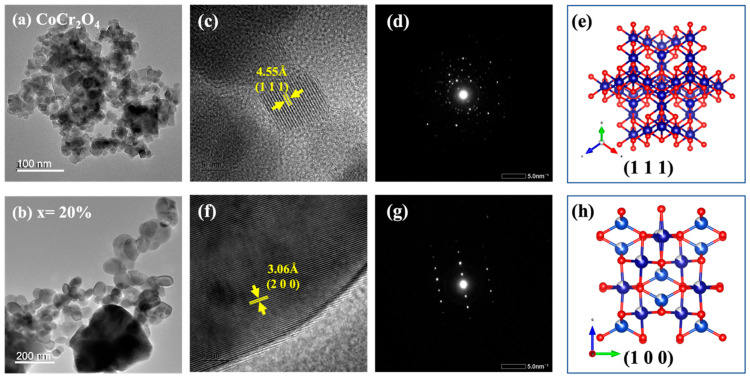
TEM and HRTEM analysis of Cu*_x_*Co_1−*x*_Cr_2_O_4_ nanoparticles. (**a**,**b**) TEM images for *x* = 0% and *x* = 20%, showing spherical particles with average sizes of 19 ± 3 nm and 55 ± 3 nm. (**c**,**f**) HRTEM images showing lattice fringes of 4.55 Å (111) for *x* = 0% and 3.06 Å (200) for *x* = 20%, indicating structural distortion from Cu doping. (**d**,**g**) SAED patterns confirm the polycrystalline nature of both samples. (**e**,**h**) Structural models highlighting (111) and (100) planes, respectively, consistent with observed lattice orientations and doping effects.

Transmission electron microscopy (TEM) and high-resolution TEM (HRTEM) are powerful techniques used to investigate the detailed structural characteristics of nanoparticles at the atomic and nanometer scales and examine the morphology and lattice structure of Cu*_x_*Co_1−*x*_Cr_2_O_4_ nanoparticles. Figure 3a,b show TEM images of samples with *x* = 0 and 20% of Cu*_x_*Co_1−*x*_Cr_2_O_4_ nanoparticles, showing nearly spherical particles with mean diameters of 19 ± 3 nm and 55 ± 3 nm, respectively. These values are in excellent agreement with XRD-derived sizes (20.6 nm and 53 nm), confirming the reliability of crystallite size estimation.

As shown in Figure 3c, the HRTEM image indicates clear lattice fringes with an interplanar spacing of 4.55 Å, corresponding to the (111) plane of the CoCr_2_O_4_ spinel structure. The selected area electron diffraction (SAED) pattern (Figure 3d) exhibits distinct diffraction spots arranged in rings, confirming the polycrystalline nature of the CoCr_2_O_4_ nanoparticles. The schematic plot represents the cubic spinel structure with the (111) plane orientation highlighted, as shown in Figure 3e. Figure 3f reveals considerable agglomeration with even larger particle sizes for the *x* = 20% Cu-doped sample. The HRTEM image displays lattice fringes with an interplanar spacing of 3.06 Å, associated with the (200) plane, confirming extensive Cu doping. The SAED pattern (Figure 3g) shows well-defined diffraction spots, confirming the retention of the polycrystalline structure. The schematic plot illustrates the (100) plane orientation, as shown in Figure 3h, reflecting significant structural modifications due to the high Cu content. This analysis indicates the systematic incorporation of Cu into the CoCr_2_O_4_ lattice, resulting in changes in the lattice parameters and the maintenance of the overall polycrystalline cubic spinel structure across varying doping levels.

### 3.4. Raman Analysis of Cu_x_Co_1−x_Cr_2_O_4_ Nanoparticles

**Figure 4:**
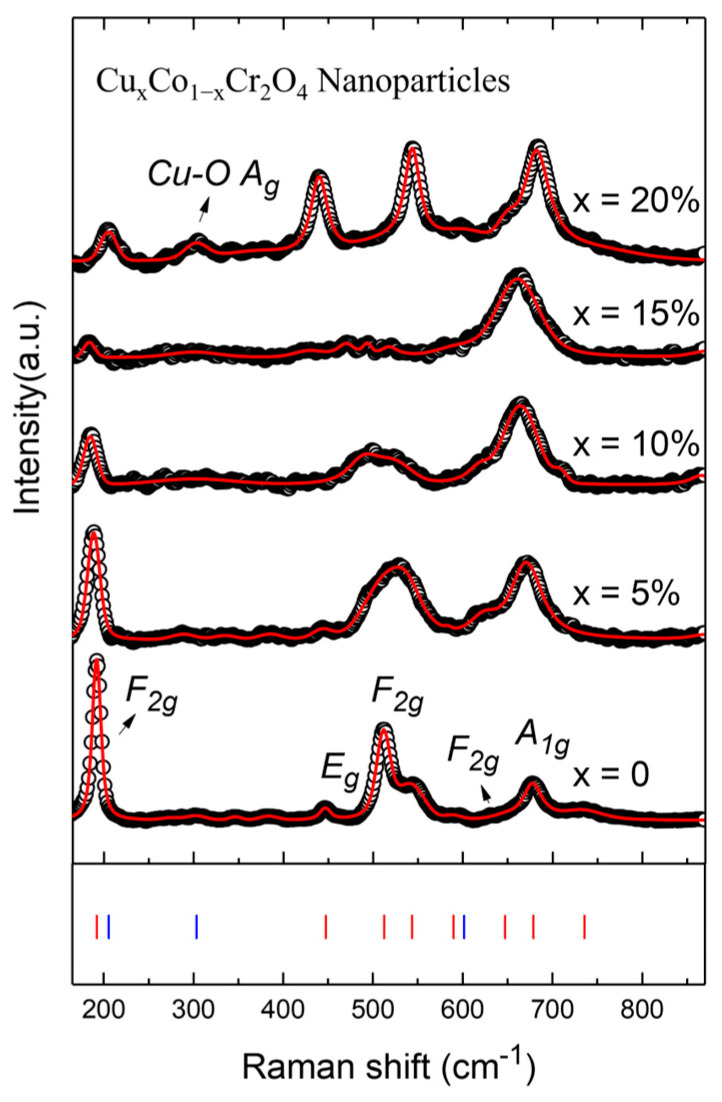
Raman spectra of various Cu*_x_*Co_1−*x*_Cr_2_O_4_ (*x* = 0, 5, 10, 15, and 20%) nanoparticles.

Raman spectroscopy serves as a powerful, non-destructive tool for probing local structural changes, symmetry distortions, and phase transitions in oxide materials. In this study, Raman scattering was utilized to examine the evolution of vibrational modes in Cu*_x_*Co_1−*x*_Cr_2_O_4_ (*x* = 0, 5, 10, 15, and 20%) nanoparticles, offering key insights into the effects of Cu doping on lattice symmetry and phase stability. The Raman spectra of all samples are shown in Figure 4. For undoped CoCr_2_O_4_, the structure adopts a normal cubic spinel phase with space group *Fd*-3*m*, which yields Raman-active modes at the Γ-point according to group theory [28]:

(2) $$\Gamma_{Fd-3m}=A_{1g}+E_{g}+{3F}_{2g}$$

These correspond to five principal peaks observed in the spectra of the pristine sample, 192 cm^−1^ ($F_{2g}$), 447 cm^−1^ ($E_{g}$), 512 cm^−1^ and 544 cm^−1^ ($F_{2g}$), and 679 cm^−1^ ($A_{1g}$), consistent with earlier reports [2,28,29]. In the 5% and 10% Cu-doped samples, these peaks remain visible with minor broadening and intensity variation, indicating that the spinel structure is largely retained. However, starting from 15% Cu doping, significant spectral changes emerge. The peaks become broader and less distinct, suggesting the onset of a structural phase transition and the coexistence of multiple crystallographic phases.

At 20% Cu doping, a clear transformation is evident. The Raman spectrum shows a dominant, broad band around 300 cm^−1^, attributed to Cu–O stretching vibrations, signaling a new bonding environment [30]. According to XRD results and supported by Raman selection rules, this transformation corresponds to a shift from the *Fd*-3*m* cubic spinel phase to a lower-symmetry *I*-42*d* tetragonal phase. For the *I*-42*d* space group, the vibrational representation is as follows:

(3) $$\Gamma_{I-42d}=A_{1g}+E_{g}+{2A}_{2u}+{2E}_{u}$$

Here, only the $A_{1g}$ and $E_{g}$ modes are Raman-active, which matches the suppression and broadening of the higher-frequency modes in the 20% doped sample. The broad peaks and frequency shifts confirm the collapse of cubic symmetry and the formation of a distorted structure with modified local coordination. The Raman spectral evolution provides strong evidence for a doping-induced phase transition driven by Cu^2+^ substitution. At intermediate doping levels (*x* = 15%), the system likely enters a biphasic regime, with both cubic and emerging lower-symmetry phases coexisting. At higher doping (*x* = 20%), the structure stabilizes into a new phase characterized by distinct vibrational signatures.

For Cu-rich compositions (*x* ≥ 0.15), the cooperative Jahn–Teller instability drives the parent *Fd*-3*m* lattice toward a tetragonal *I*4_1_*/amd* metric. Group-theoretical analysis predicts that this symmetry reduction (D_4h_⊂O_h_) splits the triply degenerate F_2g_(3) vibration of the cubic phase and renders one component Raman-active. Experimentally, we detect this as a broad shoulder centered at 620.1 cm^−1^ (*x* = 15%) (Figure 4), absent in pristine CoCr_2_O_4_. Simultaneously, the A_1g_ (Cr–O) stretching mode red-shifts from 678.7 (*x* = 0) to 662.1 cm^−1^ (*x* = 15%), and its full-width at half-maximum doubles (12 → 24 cm^−1^), confirming that Cu doping perturbs the local octahedral field, consistent with our XRD refined lattice parameters and the emergence of the Cu–O A_g_ shoulder near 301~303 cm^−1^. The linearity of the A_1g_ peak shift with *x* (≈ –1.1 cm^−1^ per at.% Cu) can be used as a quick Raman gauge for Cu content in future batches or diffusion studies, underscoring the strong lattice–phonon coupling. These observations parallel those reported for bulk CuCr_2_O_4_ [27], who attribute a comparable 610–620 cm^−1^ shoulder and 15 cm^−1^ A_1g_ shift to a static JT elongation of 0.35 Å. Thus, our Raman data provide direct, mode-specific evidence that Cu^2+^ substitution activates the same cooperative distortion pathway in the nanoparticle regime and corroborate the symmetry breaking inferred from XRD peak asymmetry.

In the study, Raman spectroscopy clearly reveals that Cu doping alters the vibrational dynamics and crystal symmetry of CoCr_2_O_4_ nanoparticles. The transition from the *Fd*-3*m* cubic spinel phase to a lower-symmetry *I*-42*d* phase is observed through peak broadening, shifts, and the emergence of new Raman modes. These structural modifications are consistent with XRD results and have significant implications for the electronic, magnetic, and magnetocaloric behavior of the material.

### 3.5. Magnetic Behavior and Doping-Driven Transitions in Cu_x_Co_1−x_Cr_2_O_4_ Nanoparticles

**Figure 5:**
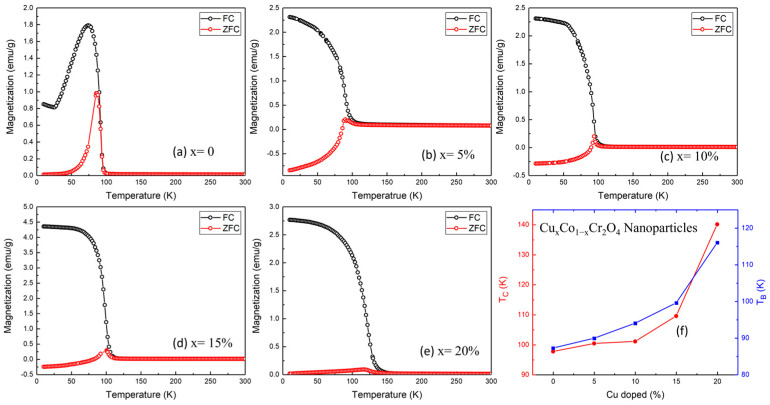
(**a**–**e**) Temperature-dependent magnetization of various Cu*_x_*Co_1−*x*_Cr_2_O_4_ (*x* = 0, 5, 10, 15, and 20%) nanoparticles. (**f**) Variation in Curie temperature *T_C_* and blocking temperature *T_B_* with Cu concentration.

To investigate magnetic behavior and transition mechanisms in Cu-doped CoCr_2_O_4_, comprehensive dc-magnetization and isothermal hysteresis measurements were performed. These include temperature-dependent magnetization (M–T) curves under zero-field-cooled (ZFC) and field-cooled (FC) conditions at an applied field of 200 Oe and magnetization vs. field (M–H) loops at 10 K, 90 K, and 300 K. Figure 5a–e display the M–T curves of all samples. The undoped sample (*x* = 0) exhibits a sharp rise in magnetization below approximately 97.74 K, marking the Curie temperature (*T_C_*) where the system transitions from a paramagnetic (PM) to a ferrimagnetic (FIM) state [29,30]. Additionally, a broad anomaly appears around 87.27 K, corresponding to the blocking temperature (*T_B_*), which signals the onset of spin freezing or anisotropic domain behavior, often attributed to the coexistence of canted spin structures or surface effects in nanosized particles. As the Cu content increases, both *T_C_* and *T_B_* exhibit a systematic upward trend, as summarized in Table 2 and plotted in Figure 5f. Specifically, *T_C_* increases from 97.74 K (*x* = 0) to 140.15 K (*x* = 20%), while T_B_ shifts from 87.27 to 116.02 K over the same range (Figure 5f and Table 2). This behavior reflects a strengthening of magnetic interactions induced by Cu doping, possibly due to enhanced superexchange between Cu^2+^–O^2−^–Cr^3+^ or the modification of cationic distribution in A/B sites of the spinel lattice.

**Figure 6:**
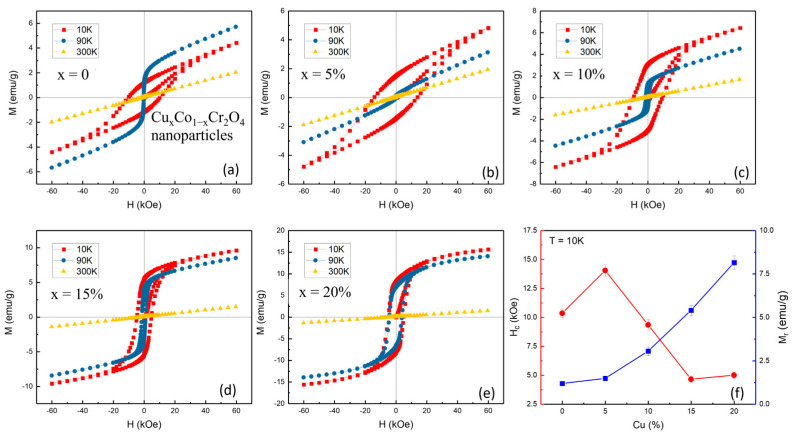
(**a**–**e**) M-H loops at 10, 90, and 300 K for Cu*_x_*Co_1−*x*_Cr_2_O_4_ (*x* = 0, 5, 10, 15, and 20%) nanoparticles. (**f**) Variation of coercivity (*H_C_*) and remanent magnetization (*M_r_*) at 10 K for Cu*_x_*Co_1−*x*_Cr_2_O_4_ nanoparticles.

The increasing bifurcation between FC and ZFC curves at higher doping levels further indicates enhanced magnetic anisotropy and possible spin-glass-like freezing or domain wall pinning, particularly visible in the *x* = 15 and 20% samples. To further assess the impact of Cu doping on the magnetic behavior of CoCr_2_O_4_ nanoparticles, magnetization versus applied field (M–H) measurements were conducted at 10, 90, and 300 K for all doping levels (*x* = 0 to 20%), as presented in Figure 6a–e. The hysteresis loops offer critical insight into the magnetic ordering, coercivity, remanent magnetization, and evolution of ferromagnetic behavior with both temperature and composition.

At 10 K, all samples display pronounced ferromagnetic (FIM) hysteresis loops [31], but with distinct variations in coercivity (*H_C_*) and remanent magnetization (*M_r_*), depending on the Cu content. The undoped sample (*x* = 0) exhibits a wide hysteresis loop with significant coercivity and high *M_r_*, characteristic of strong ferromagnetic ordering and domain wall pinning at a low temperature. As Cu is introduced at *x* = 5%, the loop becomes broader and the coercivity increases, suggesting that Cu^2+^ ions locally enhance anisotropy or pinning centers in the spinel structure. For *x* = 10%, the coercivity and loop width decrease noticeably, indicating a weakening of ferromagnetic coupling, possibly due to disorder or partial magnetic dilution caused by excess Cu^2+^. At *x* = 15 and 20%, the loops are narrower still, with reduced *H_C_* and *M_r_*, reflecting a trend toward magnetically softer behavior and reduced long-range ferrimagnetic interaction. At 90 K, the M–H loops for all samples shrink, consistent with the onset of thermal fluctuations that suppress long-range order. However, hysteresis remains visible in low-to-high doping (*x* = 0 to 20%), indicating ferrimagnetic (FM) behavior near the transition temperature. At 300 K, all compositions exhibit nearly linear M–H curves, confirming their paramagnetic nature above T_C_.

Figure 6f plots the variation of *H_C_* and *M_r_* with increasing Cu content at 10 K. Coercivity H_C_ peaks at *x* = 5%, suggesting that small Cu doping introduces localized anisotropic centers or exchange pinning, which enhances resistance to magnetization reversal. With further doping (*x* > 5%), H_C_ decreases significantly, likely due to enhanced structural disorder, spin canting, or the emergence of non-magnetic Cu^2+^ ions at critical lattice positions, disrupting coherent exchange interactions. Saturation magnetization M_S_ shows a consistent decline with Cu doping. This reduction is attributed to the partial replacement of Co^2+^ (magnetic, 3d^7^) with Cu^2+^ (magnetic but weaker exchange, 3d^9^), which modifies the A–B superexchange interactions crucial to ferrimagnetic ordering in the spinel lattice. This trend also supports the notion of magnetic dilution and a reduction in net moment, particularly due to altered spin alignment or exchange paths among Co^2+^, Cu^2+^, and Cr^3+^ cations.

Copper (Cu) doping in CoCr_2_O_4_ nanoparticles has been demonstrated to significantly modify their magnetic behavior, offering considerable promise for advanced applications. Experimental results indicate that both the Curie temperature (*T_C_*) and the blocking temperature (*T_B_*) increase with Cu incorporation, a rare phenomenon among dopants, implying that Cu^2+^ ions play an active role in reinforcing exchange interactions within the spinel lattice. Additionally, the system transitions from hard to soft magnetic behavior with increased Cu content, as evidenced by the marked decrease in coercivity (*H_C_*), aligning with prior reports of doping-induced magnetic softening [32,33,34]. At higher Cu concentrations, broadened magnetic transitions and unsaturated hysteresis loops are observed, which are attributed to enhanced spin canting, surface spin disorder, and magnetic frustration, phenomena frequently reported in doped spinel oxides. These combined effects enable fine control over magnetic parameters, making Cu_x_Co_1−x_Cr_2_O_4_ an excellent candidate for low-temperature spintronic devices, magnetic cooling systems, and sensitive magnetic sensors, where tailored coercivity and thermal response are crucial.

### 3.6. Investigation of the Magnetocaloric Effect in Cu_x_Co_1−x_Cr_2_O_4_ Nanoparticles

**Figure 7:**
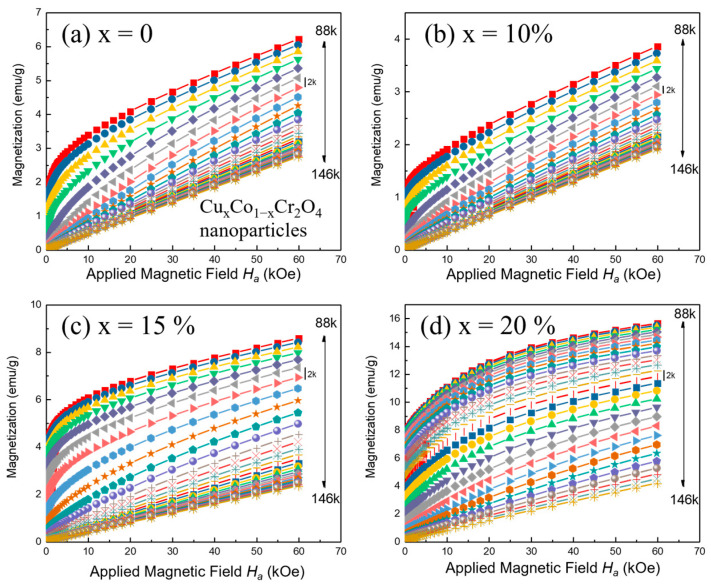
(**a**–**d**) Magnetization versus applied magnetic field (*H_a_*), measured at different temperatures with a step of 2 K for Cu*_x_*Co_1−*x*_Cr_2_O_4_ (*x* = 0, 10, 15, and 20%) nanoparticles.

To investigate the magnetocaloric effect (MCE) in Cu*_x_*Co_1−*x*_Cr_2_O_4_ nanoparticles, field-dependent isothermal magnetization measurements were performed across a series of samples with doping levels *x* = 0, 5, 10, 15, and 20%. Measurements were carried out in an applied magnetic field range of 0 to 60 kOe, with a temperature increment of 2 K, both below and above the respective Curie temperatures (*T_C_*), as shown in Figure 7a–d.

All samples demonstrated ferromagnetic (FM) behavior below their *T_C_*, evidenced by a characteristic increase in magnetization with the applied magnetic field. This response is due to the alignment of magnetic moments along the external field direction, reflecting strong magnetic interactions within the spinel lattice [17]. The resulting S-shaped M–H loops confirm the presence of ferromagnetic order. As the temperature increases beyond *T_C_*, the alignment of magnetic moments weakens due to thermal agitation, leading to induced paramagnetism and a linear magnetization response, which reflects the transition to the paramagnetic (PM) state [35]. This trend is consistent across all compositions and becomes particularly evident in the M–H curves at 90 K (as can be seen in Figure 6a–e).

The evolution from FM to PM behavior highlights the presence of a magnetocaloric effect, as the temperature-dependent magnetic ordering plays a central role. Two main features characterize this transition: 1. Spin Alignment Below *T_C_*: In the FM region, spins align readily with the applied magnetic field, producing significant magnetization and demonstrating strong ferrimagnetic interactions. 2. Thermal Disruption Above *T_C_*: As the temperature increases, thermal fluctuations disrupt spin ordering, reducing magnetization and transitioning the system to a paramagnetic regime where induced magnetization is linear with the field. The smooth and continuous transformation of magnetization with temperature and field suggests a second-order nature in the magnetic phase transition. This is further supported by the absence of hysteresis in the magnetization curves during both the magnetization and demagnetization processes, confirming the reversibility of the magnetic response.

### 3.7. Analysis of Magnetization and Arrott Plots for Cu_x_Co_1−x_Cr_2_O_4_ Nanoparticles

**Figure 8:**
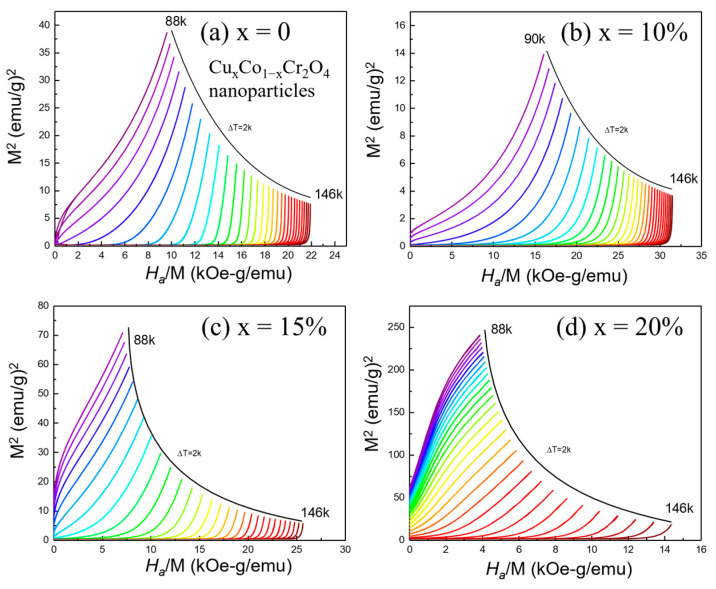
(**a**–**d**) Arrott plots at different temperatures with a step of 2 K for Cu*_x_*Co_1−*x*_Cr_2_O_4_ (*x* = 0, 10, 15, and 20%) nanoparticles.

To confirm the thermodynamic order of the transition, Arrott plots (*M*^2^ vs. *H_a_/M*) were constructed from the isothermal magnetization data (Figure 8a–d). According to Banerjee’s criterion [36], the slope of these plots determines the nature of the magnetic phase transition: a positive slope indicates a second-order phase transition (SOPT), while a negative slope is indicative of a first-order transition. The Arrott plots for all Cu-doped CoCr_2_O_4_ samples exhibit consistently positive slopes across the full temperature range, clearly supporting the classification of the PM–FIM transition as second-order. These plots also enable the precise determination of the Curie temperature (*T_C_*) by identifying the isotherm that passes linearly through the origin. As doping increases, the curvature and slope of the Arrott plots shift, indicating that Cu incorporation systematically enhances *T_C_*. These plots provide valuable information about magnetic interactions. The persistence of positive slopes across all compositions, even with substantial doping, implies that Cu^2+^ substitution maintains the cooperative nature of magnetic interactions. While Cu^2+^ ions (3d^9^) possess a weaker magnetic moment than Co^2+^ (3d^7^), their incorporation likely modifies the superexchange pathways and spin structure in a manner that supports ferrimagnetic order. The combination of field-dependent isothermal magnetization and Arrott plot analysis reveals that Cu doping induces a robust magnetocaloric response and preserves second-order phase transition characteristics. These findings are crucial in understanding and optimizing the magnetic performance of Cu_x_Co_1−x_Cr_2_O_4_ nanoparticles in magnetocaloric and other temperature-sensitive magnetic applications.

### 3.8. Assessment of Magnetic Refrigeration Efficiency in Cu_x_Co_1−x_Cr_2_O_4_ Nanoparticles

Our study explored the magnetocaloric effect (MCE) in Cu*_x_*Co_1−*x*_Cr_2_O_4_ nanoparticles synthesized with varying Cu concentrations (*x* = 0, 5, 10, 15, and 20%). The objective was to determine the impact of Cu doping on the MCE and identify the optimal concentration for maximum magnetic entropy change, a crucial parameter for magnetic refrigeration applications.

To begin with, we performed isothermal magnetization measurements across a range of temperatures (below and above the Curie temperature, T_C_) and applied magnetic fields (up to 60 kOe). These measurements allowed us to construct the M-H curves for calculating the magnetic entropy change (−Δ*S_M_*). The integral provided above was used to derive −Δ*S_M_* from the slopes of the M-H curves at different temperatures. To assess the efficiency of our sample in magnetic refrigeration systems, we utilized a series of isothermal M-H curves to calculate the magnetic entropy change (−Δ*S_M_*) induced by applying a magnetic field H. This numerical approximation measures the effectiveness of our sample in magnetic refrigeration systems. The magnetic entropy change (−Δ*S_M_*) is given by the following integral [37,38]:

(4) $$-\Delta S_{M}=\int_{0}^{H_{max}}{\left(\frac{dM}{dT}\right)}_{H}dH$$

**Figure 9:**
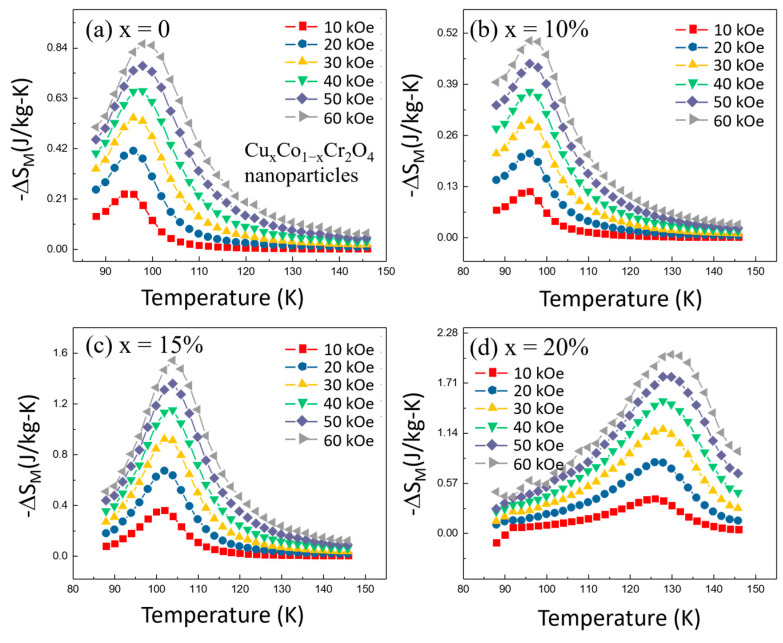
(**a**–**d**) The change in magnetic entropy varies with temperature at various applied magnetic fields for Cu*_x_*Co_1−*x*_Cr_2_O_4_ (*x* = 0, 10, 15, and 20%) nanoparticles.

The temperature dependence of the magnetic entropy change (−Δ*S_M_*) was analyzed to understand how Cu doping influenced the magnetocaloric properties. Figure 9a–d show that for all Cu-doped samples, the −Δ*S_M_* values initially increase with temperature, reaching a peak at the Curie temperature (*T_C_*). This behavior is expected, as the MCE is most pronounced near *T_C_*, where the magnetic ordering changes rapidly with temperature. Beyond *T_C_*, the −Δ*S_M_* values decrease with further increases in temperature due to the reduced magnetic interactions at higher temperatures. One of the significant findings from our study is that the magnitude of the magnetic entropy change (−Δ*S_M_*) is strongly dependent on the applied magnetic field. For each temperature, higher magnetic fields resulted in larger −Δ*S_M_* values. This trend is consistent across all doping concentrations, indicating that the MCE in Cu*_x_*Co_1−*x*_Cr_2_O_4_ nanoparticles can be effectively tuned by adjusting the strength of the external magnetic field.

Among the various Cu concentrations studied, the sample with *x* = 20% exhibited the highest peak in −Δ*S_M_* at the Curie temperature. This suggests that *x* = 10% Cu doping optimizes the magnetocaloric effect in CoCr_2_O_4_ nanoparticles. This sample’s maximum ($-∆S_{M}^{Max}$) value was significantly higher than that of the undoped sample (*x* = 0), highlighting the beneficial impact of Cu doping in enhancing the MCE. The field-dependent measurements revealed that the maximum ($-∆S_{M}^{Max}$) values increase monotonically with increasing magnetic field strength, as summarized in Table 3. For instance, at 60 kOe, the $-∆S_{M}^{Max}$ = 2.015 J/kg-K values for the *x* = 20% sample were approximately 5.57 times higher than those at 10 kOe ($-∆S_{M}^{Max}$ = 0.362 J/kg-K). This indicates that higher magnetic fields can further amplify the magnetocaloric response, making these materials more effective in practical refrigeration applications.

To provide a robust assessment of magnetocaloric performance, we compared the results of our Cu-doped CoCr_2_O_4_ nanoparticles with those of other reported CoCr_2_O_4_-based systems and related materials. As summarized in Table S1 (Supporting Information), the Cu_0.2_Co_0.8_Cr_2_O_4_ sample demonstrates a peak magnetic entropy change ($-∆S_{M}^{Max}$) of 0.776 J/kg-K at an applied field of 20 kOe, occurring near 126 K. This value surpasses those of various Fe- and Mn-doped CoCr_2_O_4_ compositions at similar or even higher fields [39,40]. For instance, Mn-substituted systems exhibit lower $-\Delta S_{M}$ values, ranging from 0.62 to 0.67 J/kg-K at 20 kOe [39], while the Fe-doped counterpart Co(Cr_1−*x*_Fe*_x_*)_2_O_4_ (*x* = 0.50) records only 0.27 J/kg-K, even at 70 kOe [17]. Even pure CoCr_2_O_4_ nanoparticles reach 0.87 J/kg-K but require a field as high as 60 kOe to do so [23].

In this context, the Cu-doped sample stands out for its effective entropy change at relatively moderate field strengths, offering significant cooling performance while maintaining material simplicity. Notably, the operating temperature around 140 K is particularly advantageous for applications in the precooling stages in superconducting systems and cryogenic infrared detectors, where moderate temperature drops and vibration-free operation are essential. These comparisons underscore the potential of Cu substitution in tuning the magnetostructural and entropy characteristics of CoCr_2_O_4_. The enhancement in $-\Delta S_{M}$ in a practical field range of 20 kOe makes Cu_0.2_Co_0.8_Cr_2_O_4_ a compelling candidate for mid-range cryogenic magnetic refrigeration technologies.

Our study also investigated the structural and magnetic properties of the Cu-doped CoCr_2_O_4_ nanoparticles to understand the underlying mechanisms driving the enhanced MCE. X-ray diffraction (XRD) analyses confirmed that the Cu-doped samples retained the spinel structure of CoCr_2_O_4_, with slight lattice distortions introduced by the Cu ions. Magnetic measurements revealed that Cu doping modifies the magnetic interactions within the spinel lattice, leading to changes in the Curie temperature and the overall magnetic behavior.

Specifically, Cu doping resulted in a noticeable increase in the Curie temperature *T_C_*, with the *x* = 20% composition exhibiting a *T_C_* of approximately 140.15 K, significantly higher than that of the undoped CoCr_2_O_4_ sample. This enhancement in *T_C_* is attributed to the substitution of Cu^2+^ ions for Co^2+^ ions, which modifies the superexchange interactions and strengthens the overall magnetic ordering. Although Cu^2+^ ions possess a weaker magnetic moment than Co^2+^, their incorporation leads to improved magnetic anisotropy and better spin alignment, which in turn contributes to a more pronounced magnetocaloric effect (MCE). Among all compositions, the *x* = 20% sample exhibits the highest magnetic entropy change −Δ*S_M_* and relative cooling power (RCP), highlighting its potential for use in energy-efficient magnetic refrigeration systems. Future research should focus on evaluating the long-term thermal and chemical stability of these materials, as well as exploring their scalability and integration into functional cooling devices to advance magnetocaloric-based technologies.

### 3.9. Relative Cooling Power (RCP) in Cu_x_Co_1−x_Cr_2_O_4_ Nanoparticles

It is important to note that −ΔS_M_ is not the sole factor determining a material’s suitability for magnetic refrigeration. The relative cooling power (RCP) is a crucial metric in evaluating the cooling effectiveness of a magnetic refrigerant. RCP combines the $\delta T_{FWHM}$ (full width at half maximum of the magnetic entropy change curve) and $-∆S_{M}^{Max}$ (maximum values of the magnetic entropy change). RCP represents the maximum achievable heat transfer rate in an ideal thermodynamic cycle between a refrigerator’s hot and cold compartments. The RCP value is calculated as follows [40]:

(5) $$RCP=\delta T_{FWHM}\times (-∆S_{M}^{Max})$$

**Figure 10:**
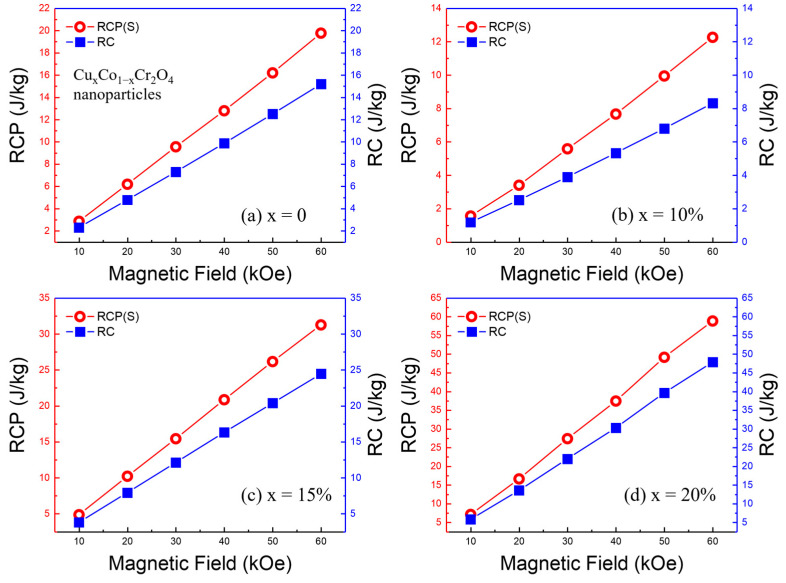
(**a**–**d**) The RCP and RC vary with the applied field for Cu*_x_*Co_1−*x*_Cr_2_O_4_ (*x* = 0, 10, 15, and 20%) nanoparticles.

At the Curie temperature (*T_C_*), the relative cooling power (RCP) of Cu*_x_*Co_1−*x*_Cr_2_O_4_ (*x* = 0, 10, 15, and 20%) nanoparticles was analyzed against the applied magnetic field. Figure 10a–d demonstrate the relationship between the absolute RCP values and the magnetic field strength, revealing a consistent increase as the field strength rises. Specifically, the maximum RCP values were approximately 19.771 J/kg for the *x* = 0 sample, 12.265 J/kg for the *x* = 10% sample, 31.262 J/kg for the *x* = 15% sample, and 58.87 J/kg for the *x* = 20% sample, all at an applied magnetic field of 60 kOe.

For context, the study of CoCr_2_O_4_ nanoparticles by Gulkesen et al. [17] reported an RCP value of 13.20 J/kg at 70 kOe, with a *T_C_* of 96 K. Additionally, the investigation of Co(Cr_0.95_Fe_0.05_)_2_O_4_ by Ram Kumar et al. [18] found an RCP value of 13 J/kg at 90 kOe, near a *T_C_* of approximately 110 K. Comparing these findings to our results, we can see that the RCP value of 58.87 J/kg at 60 kOe for the *x* = 20% sample in our study is significantly higher. This enhanced RCP value underscores the potential of our samples as effective magnetic refrigerants. The monotonic increase in RCP with the applied magnetic field highlights the robustness of the magnetocaloric effect in our Cu-doped CoCr_2_O_4_ nanoparticles. The observed trend suggests that these materials exhibit strong magnetic interactions that can be harnessed for practical cooling applications. The higher RCP values at lower applied magnetic fields than those seen in previously reported data imply that our samples could achieve efficient cooling with less energy input, making them more viable for real-world applications.

Our analysis revealed that Cu doping plays a crucial role in enhancing the magnetocaloric properties of CoCr_2_O_4_ nanoparticles. The increased RCP values with higher Cu concentrations can be attributed to several factors: Firstly, Cu doping introduces additional magnetic interactions within the spinel structure, leading to stronger magnetic coupling and an improved magnetocaloric response. Secondly, as confirmed by XRD analyses, the structural changes induced by Cu doping contribute to the enhanced magnetic entropy change, which directly impacts the RCP.

The Cu-doped samples exhibited notable changes in their magnetic and structural properties, which were systematically analyzed to understand the underlying mechanisms. Introducing Cu ions into the CoCr_2_O_4_ lattice altered the magnetic interactions, resulting in modified Curie temperatures and enhanced magnetocaloric performance. The transition from a cubic to a trigonal structure at higher Cu concentrations further amplified the magnetocaloric effect, as evidenced by the increased RCP. Moreover, the temperature dependence of the magnetic entropy change (−Δ*S_M_*) provided additional insights into the magnetocaloric behavior of the samples. The −Δ*S_M_* values increased with temperature, reaching a peak at the Curie temperature, then decreased with further temperature increases. This behavior is characteristic of materials exhibiting strong magnetocaloric effects and underscores the potential of Cu-doped CoCr_2_O_4_ nanoparticles for efficient magnetic refrigeration.

### 3.10. Refrigeration Capacity (RC) in Cu_x_Co_1−x_Cr_2_O_4_ Nanoparticles

Refrigeration capacity (*RC*) is a crucial parameter when evaluating the effectiveness of materials for refrigeration applications. *RC* represents the heat transfer between the cold and hot reservoirs in a thermodynamic cycle, providing an essential measure of the material’s cooling efficiency. The *RC* values of Cu*_x_*Co_1−*x*_Cr_2_O_4_ (*x* = 0, 10, 15, and 20%) nanoparticles can be estimated using the following equation [41]:

(6) $$RC=\int_{T_{1}}^{T_{2}}|∆S_{M}|dT$$

*T*_1_ and *T*_2_ represent the temperatures corresponding to the half-maximum values of the −Δ*S_M_* peak. As illustrated in Figure 9a–d, the *RC* values demonstrate a monotonic increase with the strength of the applied magnetic field. This trend suggests that the efficiency of the material improves with stronger magnetic fields, enhancing its effectiveness in magnetic refrigeration applications. The relationship between RC and magnetic field strength can be attributed to the improved alignment of magnetic moments under higher fields, which boosts the material’s ability to transfer heat.

The maximum *RC* values obtained are approximately 13 J/kg for the *x* = 0 sample, 8.5 J/kg for the *x* = 10% sample, 25 J/kg for the *x* = 15% sample, and 48 J/kg for the *x* = 20% sample, all at an applied magnetic field of 60 kOe. These values are significant, indicating the high potential of these nanoparticles in refrigeration applications. The achievement of such high RC values at relatively moderate field strengths underscores the efficiency of Cu*_x_*Co_1−*x*_Cr_2_O_4_ nanoparticles.

The RC values of Cu_x_Co_1−x_Cr_2_O_4_ nanoparticles compare favorably with those of other materials reported in the literature. For instance, the *RC* values obtained are higher than those described for Bi- and Mg-doped CoCr_2_O_4_ and pristine CoCr_2_O_4_ nanoparticles in previous studies [37]. This comparison highlights the advantages of Cu doping in enhancing the refrigeration capacity and overall magnetic properties of CoCr_2_O_4_. The variation in *RC* with different Cu concentrations (*x* = 0–20%) provides insights into the doping effects on the material’s performance. As the Cu concentration increases, notable changes in the magnetic properties directly influence the *RC*. Optimizing the Cu concentration is crucial in achieving the best refrigeration performance.

The detailed analysis of the refrigeration capacity (*RC*) in Cu*_x_*Co_1−*x*_Cr_2_O_4_ nanoparticles underscores their potential as efficient materials for magnetic refrigeration. The high *RC* values and the ability to tailor magnetic properties through Cu doping position these nanoparticles as strong candidates for advanced cooling applications. The high *RC* values and favorable comparison with other materials suggest that Cu*_x_*Co_1−*x*_Cr_2_O_4_ nanoparticles are promising candidates for magnetic refrigeration. The ability to tailor the material’s properties through Cu doping offers a pathway toward optimized performance for specific applications.

The monotonic increase in RC with applied magnetic field strength further supports the feasibility of these nanoparticles in practical cooling systems where strong magnetic fields can be applied. The trends and comparative advantages observed underscore the importance of continued research and development in this area to completely fulfil these materials’ potential. By leveraging the enhanced magnetic properties imparted by Cu doping, these nanoparticles could be effectively utilized in next-generation refrigeration technologies, offering improved efficiency and reduced environmental impacts. The ability to achieve high *RC* values at moderate field strengths, coupled with the observed enhancement in magnetic properties through Cu doping, positions these materials as highly promising for advanced cooling systems. Further optimization and detailed studies on their magnetic and structural properties could pave the way in developing efficient, environmentally friendly refrigeration technologies.

## 4. Conclusions

This study provides a detailed analysis of the structural, magnetic, and magnetocaloric properties of Cu-doped CoCr_2_O_4_ nanoparticles synthesized via solution combustion. The results show that Cu^2+^ substitution leads to a progressive structural transformation from a cubic spinel (*Fd*-3*m*) to a trigonal corundum (*R*-3*c*) phase at 20% doping, accompanied by increased particle size, enhanced crystallinity, and reduced agglomeration. These structural modifications, confirmed by XRD, Raman spectroscopy, and SEM, directly influence the magnetic properties.

Cu doping induces a clear enhancement in magnetic ordering, as evidenced by the increase in Curie temperature from 97.7 K (*x* = 0) to 140.2 K (*x* = 20%), and a shift in magnetic behavior from hard to soft ferrimagnetism. The magnetocaloric effect (MCE) is significantly strengthened at higher Cu concentrations, with the *x* = 20% sample achieving a peak magnetic entropy change ($-∆S_{M}^{Max}$) of 2.015 J/kg-K and a relative cooling power (RCP) of ~58.8 J/kg under a moderate magnetic field of 60 kOe. Arrott plot analysis confirms second-order phase transitions across all doping levels.

These findings establish Cu*_x_*Co_1−*x*_Cr_2_O_4_ as a tunable and efficient cryogenic magnetocaloric material. The ability to engineer the MCE response through Cu doping, without reliance on rare-earth elements, makes this system particularly attractive for low-temperature solid-state cooling. However, limitations remain; long-term stability under thermal cycling was not assessed, and performance beyond 20% doping remains unexplored. Future work should address these aspects, including co-doping strategies, chemical durability, and potential integration into functional refrigeration prototypes.

In summary, Cu-doped CoCr_2_O_4_ nanoparticles offer appreciable MCE performance near 140 K under moderate fields, suggesting their potential in cryogenic magnetic refrigeration for laboratory-scale or specialized applications.

## Figures and Tables

**Table 1:** Refined structural parameters for Cu*_x_*Co_1−*x*_Cr_2_O_4_ nanoparticles obtained from Rietveld and Williamson–Hall analyses of powder X-ray diffraction patterns. Lattice parameters (*a*) were extracted from full-pattern refinements; crystallite sizes ($\langle d_{XRD}\rangle$) and micro-strain (ε) were calculated using the Uniform Deformation Energy Density Model (UDEDM) and Williamson–Hall plots. The cation–anion hopping lengths *L_A_* (tetrahedral) and *L_B_* (octahedral) were derived from the refined lattice constant, while the elastic energy density $U=\frac{1}{2}E\varepsilon^{2}$ (with E = 200 GPa) quantifies the strain energy stored per unit volume. The data reveal a sharp micro-strain maximum and corresponding energy density peak at *x* = 0.10, marking the composition where cooperative Jahn–Teller distortion begins to dominate the spinel lattice.

x (%)	*a* (Å)	$\langle d_{XRD}\rangle$ (nm)	ε(%)	*L_A_* (Å)	*L_B_* (Å)	U (MJ/m^3^)
0	8.3212	20.6	0.239	3.6032	2.35359	0.57
5	8.3207	30.5	0.208	3.6030	2.35345	0.43
10	8.3077	30.7	0.384	3.5973	2.34977	1.48
15	8.455	36.5	0.114	3.6611	2.39144	0.13
20	8.347	53.7	0.22	3.6144	2.36089	0.48

**Table 2:** Summary of magnetic transition parameters of Cu_x_Co_1−x_Cr_2_O_4_ nanoparticles as a function of the Cu^2+^ doping concentration. The magnetic blocking temperature (*T_B_*) is obtained from the bifurcation point of zero-field-cooled (ZFC) and field-cooled (FC) magnetization curves, and the error bars are estimated from the temperature dependency of magnetization. The Curie temperature (*T_C_*) is extracted from the inflection point in the dM/dT analysis. The coercivity (*Hᴄ*) and remanent magnetization (*M_r_*) are measured at 10 K, and the error bars are estimated from the hysteresis loop.

Cu^2+^ (%)	*T_B_* (K)	*T_C_* (K)	*H_C_* (kOe)	*M_r_* (emu/g)
0	87.3 ± 0.5	97.7 ± 0.6	10.4 ± 0.4	1.2 ± 0.1
5	89.9 ± 0.6	100.4 ± 0.6	14.1 ± 0.5	1.5 ± 0.1
10	94.1 ± 0.6	101.1 ± 0.7	9.4 ± 0.5	3.1 ± 0.3
15	99.6 ± 0.6	109.6 ± 0.8	4.7 ± 0.3	5.4 ± 0.3
20	116 ± 0.7	140 ± 1.0	5.0 ± 0.3	8.2 ± 0.4

**Table 3:** Summary of maximum magnetic entropy change ($-∆S_{M}^{Max}$) and the peak temperature ($T_{C}^{Max}$) obtained from the change in magnetic entropy varies with temperature at various applied magnetic fields for Cu*_x_*Co_1−*x*_Cr_2_O_4_ (*x* = 0, 10, 15, and 20%). The results demonstrate a significant enhancement in $-∆S_{M}^{Max}$ with increasing Cu content, especially at *x* = 20%, indicating improved magnetocaloric performance.

	*x* = 0%		*x* = 10%		*x* = 15%		*x* = 20%	
H*_a_* (kOe)	$T_{C}^{Max}$ (K)	$-∆S_{M}^{Max}$ (J/kg-K)	$T_{C}^{Max}$ (K)	$-∆S_{M}^{Max}$ (J/kg-K)	$T_{C}^{Max}$ (K)	$-∆S_{M}^{Max}$ (J/kg-K)	$T_{C}^{Max}$ (K)	$-∆S_{M}^{Max}$ (J/kg-K)
10	94.17	0.229	94.32	0.112	100.84	0.342	124.81	0.362
20	95.14	0.395	94.61	0.202	101.62	0.631	125.92	0.776
30	96.01	0.527	94.93	0.281	102.26	0.875	126.85	1.133
40	96.88	0.638	95.22	0.354	102.88	1.091	127.71	1.451
50	97.73	0.736	95.52	0.421	103.45	1.283	128.54	1.731
60	98.58	0.828	95.77	0.482	103.99	1.459	129.39	2.015

## Data Availability

Data are contained within the article and Supplementary Materials.

## Supplementary Materials

The following supporting information can be downloaded at https://www.mdpi.com/article/10.3390/nano15141093/s1. Scheme S1: Schematic diagram of the combustion synthesis process for Cu doping on CoCr_2_O_4_ nanoparticles; Figure S1: EDX analysis of Cu_x_Co_1−*x*_Cr_2_O_4_ (*x* = 0, 5, 10, 15, and 20%) nanoparticles; Figure S2: Williamson–Hall plots of CuxCo_1−*x*_Cr_2_O_4_ (*x*= 0, 5, 10, 15, and 20%) nanoparticles with fit equations $\beta_{T}\cos\theta =\frac{k\lambda }{\langle d_{XRD}\rangle }+4\varepsilon \sin\theta$ that extracted crystallite size 〈d_XRD_〉, microstrain *ε*, and error bars; Table S1: Comparison of the magnetocaloric performance of Cu_0.2_Co_0.8_Cr_2_O_4_ nanoparticles (this work) with selected benchmark and literature-reported materials. The table summarizes the peak magnetic entropy change ($-\Delta S_{M}$) and the applied magnetic field range for each material under various applied magnetic fields. The data highlight the competitive performance of the present material compared to other transition-metal-based magnetocaloric systems [17,23,39].
