# Supplementary material for: Cu-Doping Induced Structural Transformation and Magnetocaloric Enhancement in CoCr2O4 Nanoparticles
Source: Nanomaterials (Basel). 2025 Jul 14;15(14):1093. doi: 10.3390/nano15141093 (PMC12300304; doi:10.3390/nano15141093)
Supplement: Supplementary file 1 [file nanomaterials-15-01093-s001.zip › nanomaterials-3695096-supplementary.pdf]

Supporting Information

Cu-Doping Induced Structural Transformation and Magnetocaloric Enhancement in $CoCr_2O_4$ Nanoparticles

Ming-Kang Ho[a, b], Yun-Tai Yu[a], Hsin-Hao Chiu[a,b], K. Manjunatha[a, *], Shih-Lung Yu[a], Bing-Li Lyu[a], Tsu-En Hsu[a], Heng-Chih Kuo[a], Shuan-Wei Yu[a], Wen-Chi Tu[a], Chiung-Yu Chang[a], Chia-Liang Cheng[a], H. Nagabhushana[c], Tsung-Te Lin[d], Yi-Ru Hsu[d], Meng-Chu Chen[e, *], Yue-Lin Huang[a], and Sheng Yun Wu[a, *]

[a]*Department of Physics, National Dong Hwa University, Hualien 97401, Taiwan*

[b]*National Synchrotron Radiation Research Center, Hsinchu 30076, Taiwan*

[c]*Prof. C.N.R. Rao Centre for Advanced Materials, Tumkur University, Tumkur 572103, India*

[d]*Department of Mechanical and Systems Engineering, National Atomic Research Institute, Taoyuan 325207, Taiwan*

[e]*Department of Applied Science, National Taitung University, Taitung 950, Taiwan*

*Corresponding author: kmanjunatha@gms.ndhu.edu.tw (KM); mchen@nttu.edu.tw (MCC); sywu@gms.ndhu.edu.tw (SYW)

Scheme S1 Schematic diagram of the combustion synthesis process for Cu doping on $CoCr_2O_4$ nanoparticles.

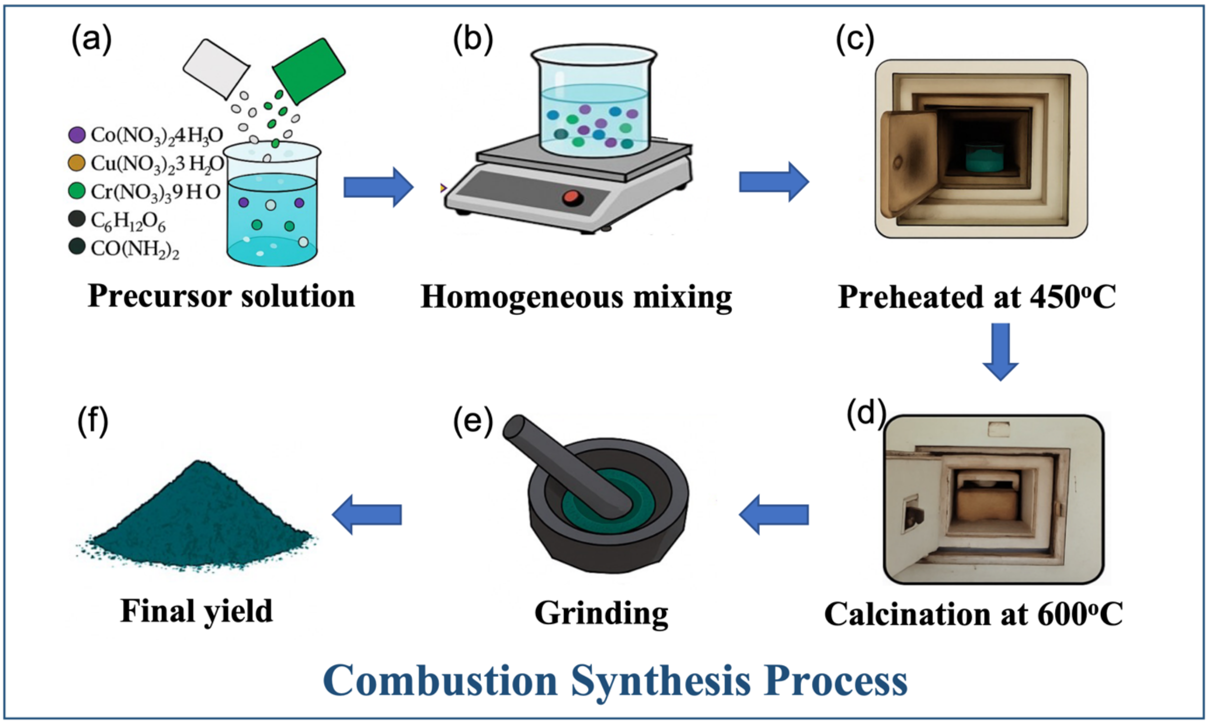

Figure S1 (a)-(e) EDX analysis of $Cu_xCo_{1-x}Cr_2O_4$ (x = 0, 5, 10, 15, and 20%) nanoparticles.

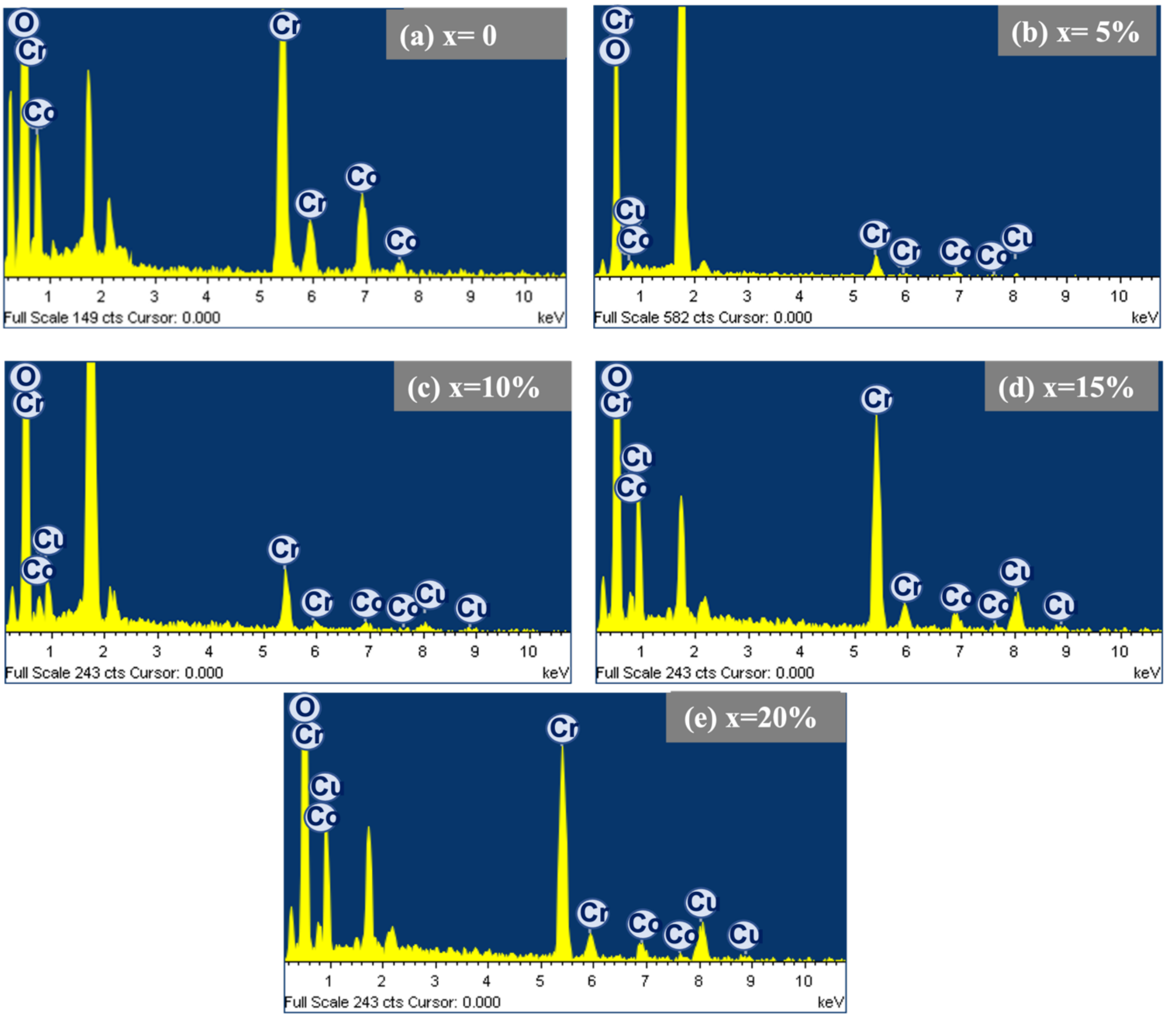

Figure S2 (a)-(e) Williamson–Hall plots of $Cu_xCo_{1-x}Cr_2O_4$ (x= 0, 5, 10, 15, and 20%) nanoparticles with fit equations $\beta_T \cos\theta = \frac{k\lambda}{\langle d_{XRD}\rangle} + 4\varepsilon \sin\theta$ that extracted crystallite size $\langle d_{XRD}\rangle$, microstrain ε, and error bars.

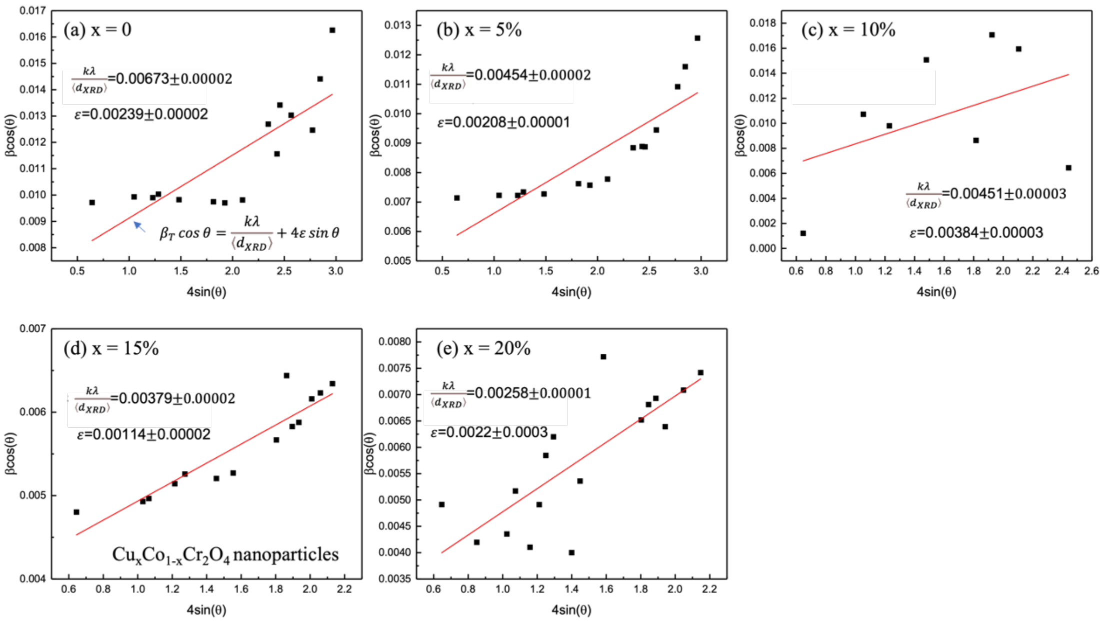

Table S1: Comparison of the magnetocaloric performance of $Cu_{0.2}Co_{0.8}Cr_2O_4$ nanoparticles (this work) with selected benchmark and literature-reported materials. The table summarizes the peak magnetic entropy change ($-\Delta S_M^{Max}$) and the applied magnetic field range for each material under various applied magnetic fields. The data highlight the competitive performance of the present material compared to other transition-metal-based magnetocaloric systems.

Materials	Peak (K)	$-\Delta S_M^{Max}$ (J/kg-K)	Reference
**$Cu_xCo_{1-x}Cr_2O_4$(x = 0.2)**	**126**	**0.776@20 kOe**	**This work**
$Co(Cr_{1-x}Fe_x)_2O_4$ (x = 0.0)	96	0.88@70 kOe	[S1]
$Co(Cr_{1-x}Fe_x)_2O_4$ (x = 0.25)	312	0.88@70 kOe	[S1]
$Co(Cr_{1-x}Fe_x)_2O_4$ (x = 0.50)	312	0.27@70 kOe	[S1]
Pure $CoCr_2O_4$ (nanoparticles)	96	0.87@60 kOe	[S2]
$Co(Cr_{1-x}Mn_x)_2O_4$ (x = 0.0)	-	0.67@20 kOe	[S3]
$Co(Cr_{1-x}Mn_x)_2O_4$ (x = 0.1)	-	0.64@20 kOe	[S3]
$Co(Cr_{1-x}Mn_x)_2O_4$ (x = 0.2)	-	0.62@20 kOe	[S3]

References

[S1] Gulkesen, S.; Tumen, K. U.; Akyol, M.; Ekicibil, A. Room-temperature magnetocaloric effect in Fe-substituted $CoCr_2O_4$ spinels. *Appl. Phys. A* **2021**, *127*, 211. https://doi.org/10.1007/s00339-021-04374-3

[S2] Ho, M.-K.; Chiu, H.-H.; Hsu, T.-E.; Chethan, B.; Yu, S.-L.; Jheng, C.-Y.; Chin, C.-E.; Selvam, R.; Angadi, J. A. V.; Cheng, C.-L.; Nagabhushana, H.; Manjunatha, K.; Wu, S. Y. Advancing humidity sensing and magnetocaloric properties of spinel structural $CoCr_2O_4$ nanoparticles achieved via innovative bismuth doping by combustion synthesis. *Mater. Today Chem.* **2024**, *35*, 101907. https://doi.org/10.1016/j.mtchem.2024.101907

[S3] Nadeem, K.; Rehman, H. U.; Zeb, F.; Ali, E.; Kamran, M.; Noshahi, N. A.; Abbas, H. Magnetic phase diagram and dielectric properties of Mn-doped $CoCr_2O_4$ nanoparticles. *J. Alloys Compd.* **2020**, *832*, 155031. https://doi.org/10.1016/j.jallcom.2020.155031
